# Assessing physicians’ agreement and the completeness of the decision aid ‘arriba Diabetes’: a cross-sectional study

**DOI:** 10.1186/s12875-025-02887-x

**Published:** 2025-07-08

**Authors:** Nicole Lindner, Marie-Christine Hoffmann, Jörg Haasenritter, Jan K. Woike, Norbert Donner-Banzhoff

**Affiliations:** 1 Department of Primary Care, University of Marburg, Marburg, Germany; 2 School of Psychology, University of Plymouth, Plymouth, UK; 3 Centre for Adaptive Rationality, Max Planck Institute for Human Development, Berlin, Germany

**Keywords:** Diabetes mellitus, type 2 (MeSH), Deciscion support systems (MeSH), Patient-centered care (MeSH), Shared-decision-making, Personalized medicine, Diabetes software tool

## Abstract

**Background:**

Guidelines for type 2 diabetes emphasise individualised treatment goals, yet implementation remains challenging in primary care. To address this, we developed the “arriba Diabetes” software, a patient-centred decision support tool. The software provides individualised recommendations for intensity of treatment based on four inputs: age, comorbidities, treatment burden preference, and risk reduction preference.

**Methods:**

In a cross-sectional evaluation study in German primary care, we included 34 general practitioners (GPs) and 152 patients. The primary aim of this study was to evaluate the “arriba Diabetes” software by assessing the agreement between its treatment intensity recommendation and physicians’ clinical judgement. Additionally, we explored the cases of disagreement, the distribution of patient-specific inputs and physicians’ perspectives on the software’s usability.

**Results:**

The “arriba Diabetes” recommendations aligned in 87% of cases with GPs’ recommendation, and 87% of the doctors would use “arriba Diabetes” in the future. Patients had a median age of 68 years with a low comorbid load (median 3 on a scale 0–10). Patients expressed a moderate preference for higher treatment burden (median 6 on a scale 0–10) and a high preference to reduce organ complications in the future (median 8 on a scale 0–10). Acceptance of therapy burden correlated positively with the preference to reduce organ damage (Spearman correlation coefficient: +0.49).

**Conclusions:**

Recommendations of “arriba Diabetes” were well aligned with GPs’ recommendations. Implementation of the “arriba Diabetes” software has the potential to promote patient-centred and evidence-based diabetes treatment decisions in primary care.

**Graphical Abstract:**

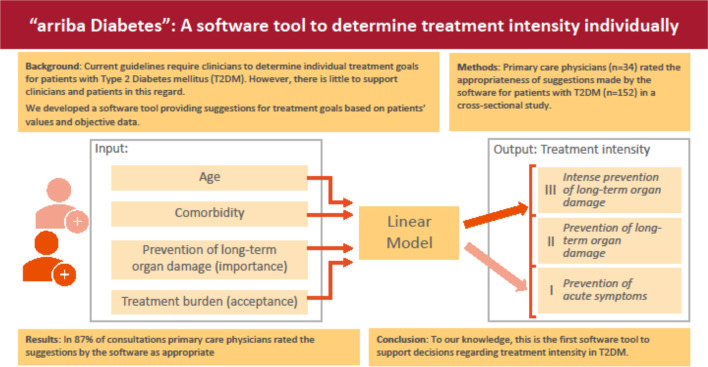

**Supplementary Information:**

The online version contains supplementary material available at 10.1186/s12875-025-02887-x.

## Background

Guidelines and statements on diabetes care require the determination of treatment goals and targets [1, 2]. These goals should be individualised; a “one size-fits all approach” is regarded as inappropriate. Despite this, guidelines have little to say on how clinicians should achieve this objective. As a result, physicians and patients often feel frustrated by an apparently impossible task [3].

Decision aids have been developed to help patients express their preferences according to their values. The utilization of evidence-based decision aids in primary care and diabetes care in particular has demonstrated effectiveness regarding various outcomes, such as reducing decisional conflict and enhancing overall satisfaction with the decisions made [4, 5]. Previous decision aids, however, addressed life-style change and specific treatment choices [6–8]. To close the gap concerning treatment goals in type 2 diabetes, we have developed “arriba Diabetes”.

“arriba Diabetes” belongs to a well-established family of software solutions to be used during primary care consultations. In various contexts such as cardiovascular prevention and managing depression, the “arriba” software has shown to be effective [9]. “arriba” follows a patient-centred philosophy addressing patient’s individual preferences, needs and values [10].

The aim of this study was to provide a first evaluation of the newly developed “arriba Diabetes” software. Specifically, we assessed the agreement between the treatment intensity suggested by the software and the physicians’ clinical judgement for each patient. Additionally, we examined the distribution of input variables, explored cases of disagreement, and gathered physician feedback on the software’s usability and potential for future implementation.

## Methods

The study followed the Declaration of Helsinki [11]. We obtained ethical approval from the Ethic Board of the University of Marburg (AZ 91/22). Members of the patient advisory board of the Department of Primary Care supported the study. The study process underwent an internal peer review by the entire working group of the Department of Primary Care (Marburg) and preliminary results were presented at the German Primary Care Congress.

### Development of “arriba Diabetes”

The development of the software broadly followed the UK Medical Research framework for developing and evaluating complex interventions and the International Patient Decision Aids Standards [12, 13]. Here we report the results of a cross-sectional evaluation study. Qualitative components of the evaluation process will be published separately.

As basis for the software prediction, we specified a linear model with four inputs:

1. age,
2. comorbidity,
3. treatment burden, and.
4. prevention of long-term organ damage.

The following considerations were the basis for the selection of these four inputs: (1) age and (2) comorbidity are suggested as relevant for the determination of treatment goals by most guidelines [1, 2, 14]. The importance of (3) treatment burden is underlined by economic analyses [15–17]. We consider (4) preference regarding the prevention of long-term organ damage as important, because most diabetes treatment efforts are geared towards this goal, as opposed to short-term symptom control. This dimension, however, was not covered by the other three. As the study by the Ann Arbor Group was conducted before the availability of recent drug classes to be taken orally (gliptins, SGLT2-inhibitors) and/or with less risk of hypoglycaemia in comparison to insulin, we reduced the weight of the third input (treatment burden) to 0.8, while the other inputs were equally weighted as ‘1’ [15–17]. This adjustment reflects the evolving treatment landscape and the relatively lower burden associated with newer therapeutic options. The decision to reduce the weight of treatment burden was informed by expert opinion. The evaluation of the underlying linear model is the primary focus of this study. The output is a single, continuous variable denoting the treatment intensity guiding the management of the patient.

During development, we discussed our model with experts in the field of diabetes and with the local patient advisory board. We incorporated the feedback received and adapted the model accordingly.

### “arriba Diabetes” software

We report “arriba Diabetes” according to the template for intervention description and replication (TIDieR) checklist [18].

“arriba Diabetes” offers training material for physicians and a leaflet for patients to prepare for the consultation. Hereby patients are encouraged to reflect on two areas:

- To what degree they accept treatment burden, such as control visits, one or more drugs to be taken regularly, injections, glucose self-measurements, behavioural change etc.
- How important they feel is the prevention of long-term organ-damage, including microvascular (eye, kidney, peripheral nervous system) and macrovascular (coronary heart disease, stroke) complications. These only partially preventable complications typically develop over years or decades. They affect only a minority of patients, depending on likelihood and individual susceptibility [19, 20].

During the consultation, physicians enter the four inputs presented above into the software: (1) age (in years), (2) a global estimate of comorbidities (0 to 10 points) followed by two preferences based on explicit discussion with the patient: accepted (3) treatment burden (0–10 points) and (4) preference regarding organ damage (0–10 points). To ensure consistency in assessing these inputs, physicians are provided with detailed training materials, including illustrative examples. Especially for input (2) global estimate of comorbidities, comprehensive training materials are important for GPs. These materials provide examples of diseases that substantially affect life expectancy and are thus relevant for the comorbidity score, such as malignancies without curative treatment options or heart failure. On the other hand examples of diseases which are not important for the score are provided, such as migraine or recurrent back pain. During the consultation, physicians explain the meaning and relevance of the preference variable, especially if the patient is unable to read the leaflet in preparation of the visit.

Based on these four inputs, “arriba Diabetes” suggests an individualized treatment intensity as a continuous variable. For easier interpretation output is divided into three levels.

i) prevention of acute symptoms of diabetes;
ii) prevention of acute symptoms of diabetes AND prevention of long-term organ damage;
iii) prevention of acute symptoms of diabetes AND *intensive* prevention of long-term organ damage.

For each level, HbA1C-limits are provided according to the age of the patient [21]. The specific HbA1C targets are presented in Table 1. The patient’s inputs and corresponding output are visually represented by bars. Physicians and patients are encouraged to critically discuss the appropriateness of the output. They can modify the inputs and observe their influence on the output. In a last step “arriba Diabetes” compares the desired level of metabolic control with the patient’s current HbA1C. Depending on the result, recommendations are given to continue as before, tighten metabolic control or check HbA1C levels again and, if appropriate, reduce glucose-lowering medication.

The consultations usually takes about 15 min. At the end of the conversation, patients receive a summary and their agreed treatment plan in a printed handout. “arriba Diabetes” can be used in newly diagnosed diabetes or in a follow-up consultation. In supplement 1 a more detailed presentation of the “arriba Diabetes” software is provided.

**Table 1:** HbA1C- targets of treatment intensity levels

Treatment intensity	Age [years]	HbA1c targets
I (prevention of acute symptoms of diabetes)	20–39	≤ 9%
	40–59	≤ 9.2%
	≥ 60	≤ 9.5%
II (prevention of acute symptoms of diabetes AND prevention of long-term organ damage)	20–39	≤ 8.0%
	40–59	≤ 8.2%
	≥ 60	≤ 8.5%
III (prevention of acute symptoms of diabetes AND *intensive* prevention of long-term organ damage)	20–39	7-7.5%
	40–59	7.2–7.7%
	≥ 60	7.5-8.0%

### Study design and setting

This cross-sectional evaluation study was conducted to assess the performance of the “arriba Diabetes” software. The study was designed to reflect real-world primary care conditions. We recruited primary care physicians in different regions throughout Germany via the “arriba community” (association of physicians using “arriba”, https://arriba-genossenschaft.de/) by e-mail and personal contact. To participate, physicians needed to provide care for patients with type 2 diabetes and have access to computer equipment. Eligible patients were approached by cooperating physicians and included if they had a diagnosis of type 2 diabetes of any duration or severity and were over 18 years old. Exclusion criteria were cognitive impairment or lack of German language skill preventing informed consent and participation. GPs approached patients during routine appointments and were supposed to conduct four consultation within a four-week period. All participants provided informed consent before participating.

### Study procedures

We provided participating physicians the “arriba Diabetes” software along with training materials which included a brochure and a short video explaining the software and its use. In case of questions, they could contact the study team. We collected sociodemographic data of participating physicians using a questionnaire. During their regular consultation hours, physicians went through the process described above. Following each consultation, physicians completed a case report form (details are presented in supplement 2), rating their agreement with the software, their likelihood of using it in practice, and their perceptions of usability. Additionally, we collected input and output data of each patient. Data underwent a deidentification procedure and clear names were replaced by pseudonyms. We managed data in Excel 2016 (Microsoft).

### Data analysis

Data analysis was performed using Rstudio 2023 (R Core Team). We calculated the proportion of consultations in which physicians agreed with the software’s recommendation, using the Exact Binomal Test (Clopper and Pearson) to determine confidence intervals. To further explore instances of non-agreement, we created visual comparisons between the software’s treatment intensity recommendations and the intensity levels that physicians considered appropriate. Additionally, we applied descriptive statistics to examine the medians and distributions of the inputs and outputs. We used Spearman’s rank correlation to assess correlations between the inputs and visualised them using a correlation heatmap. Finally, we explored physicians’ views on the software by examining free-text comments, which were summarised thematically. We used chatGPT 4.0 (OpenAI) to assist coding in R studio and to assist text editing of the manuscript.

### Sample size

For pragmatic reasons – given the exploratory nature of the study - we planned to include 120 patients recruited by 30 physicians into this early evaluation study. However, we did not limit the number of participants in each practice. The main outcome we were interested in was the agreement between intensity suggested by the software and the opinion of physicians for each patient. The appropriateness of the sample size was assessed post hoc, considering the precision of the confidence intervals.

## Results

### Participant characteristics

From September 2022 to February 2023, 34 physicians working in primary care in different regions of Germany took part in the study. Included physicians were aged 49.5 years in the median and had worked in the ambulatory setting for 10 years in the median. From September 2022 to April 2023 physicians used “arriba Diabetes” for the consultation in 152 patients. Most of the physicians used “arriba Diabetes” in four patients. Median age of patients was 68 years, 59% were male and most had type 2 diabetes for more than 10 years. Other baseline characteristics for physicians and patients can be found in Tables 2 and 3.

**Table 2:** **Sociodemographic characteristics of included physicians**

age (years) median and interquartile range	49.5 [40.25-55]
Gender absolute number and percentages	men: 22 (64.71%), women: 12 (35.3%)
years of working in ambulatory setting median and interquartile range	10 [4–21]
working pattern absolute number and percentages	full-time: 28 (82.35%), part-time: 6 (17.65%)
type of surgery absolute number and percentages	single practice: 7 (20.59%), group practice: 27 (79.4%)
location of practice absolute number and percentages	urban: 14 (41%), rural: 19 (56% ), 2 locations: 1 (3% )

**Table 3:** **Sociodemographic characteristics of included patients.**

age (years) median and interquartile range	68 [60–74] ng: 3
Gender absolute number and percentages	men: 90 (59.21%), women: 61 (40.13%), ng: 1 (0.66%))
duration of diabetes (years) absolute number and percentages	< 5 years: 38 (25%) 5–10 years: 43 (28.29%) > 10 years: 70 (46.05%) ng: 1 (0.66%)

ng: not given

### Physicians’ views on the “arriba Diabetes” recommendation

“arriba Diabetes” recommendation was well aligned with the physicians’ view on therapy intensity: In 87% (95% confidence interval: 80.4–91.8%) of the consultations, the physician felt that the recommendation of the software was appropriate for their patient.

**Fig. 1:**
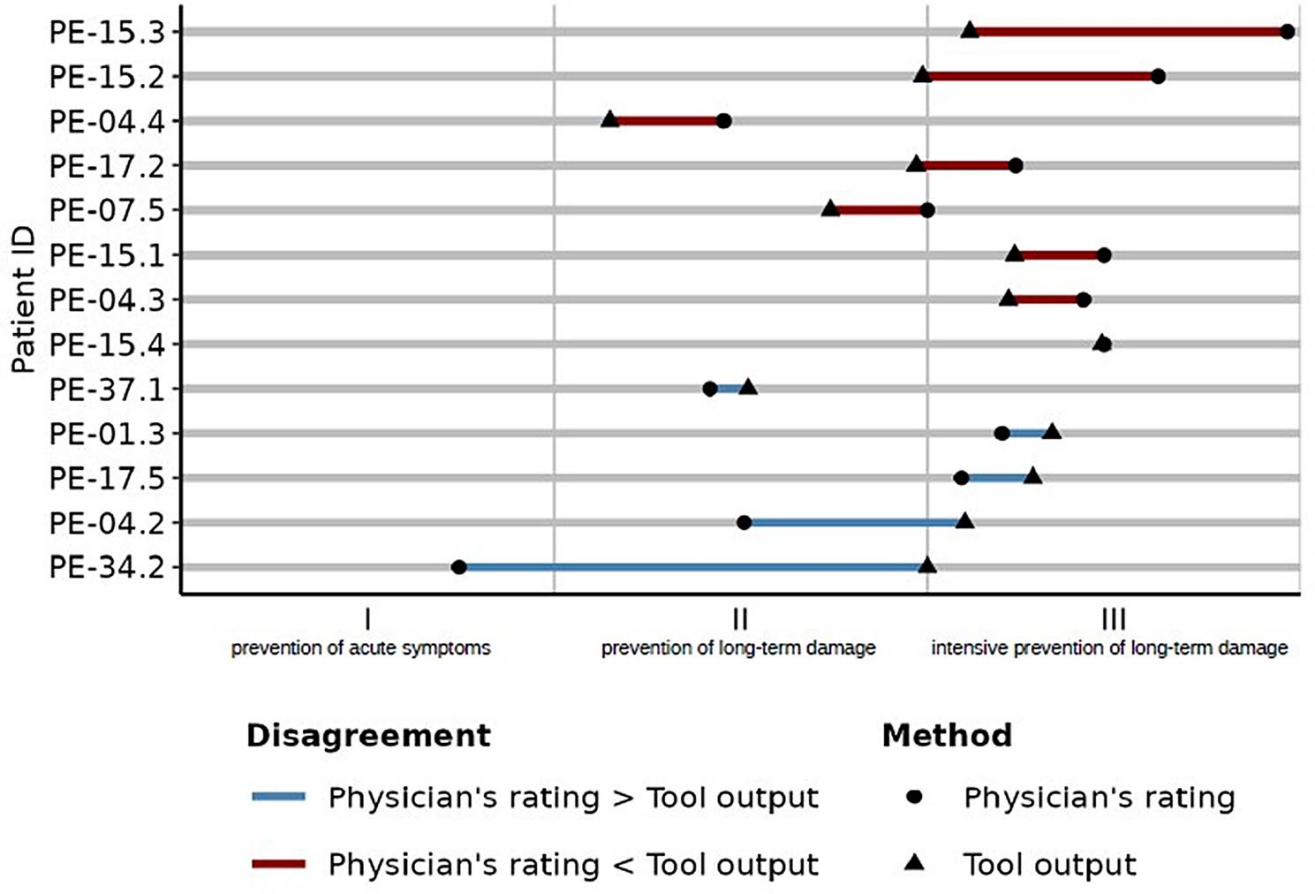
Comparison of physician and “arriba Diabetes” recommendations for cases with discrepancy: The figure illustrates the discrepancies between physician recommendations regarding therapy intensity in three categories and those generated by “arriba Diabetes”. In 19 out of 152 consultations the recommendation was not felt as appropriate. 13 physicians provided their own recommendation shown in the picture (the remaining six consultation were excluded due to missing data)

In the 19 cases where disagreement occurred, GPs provided their judgement regarding treatment intensity in 13 instances and we explored the cases where the recommendation of “arriba Diabetes” was not regarded appropriate. In these cases, the direction of disagreement was symmetrically distributed: In 8 cases physicians leaned towards favouring a tighter metabolic control, while in 5 cases physicians leaned towards a more lenient approach. In 8 cases, the level of disagreement was relatively small, and the HbA1c range was still seen as appropriate. Only in 5 cases the different judgement would have resulted in a different therapy intensity category with a different HbA1C target. In Fig. 1 a graphical presentation of cases, where the recommendation was not perceived as appropriate, is presented.

### Physicians’ willingness to use “arriba Diabetes” in clinical practice

Nearly all doctors indicated that they would use “arriba Diabetes” in future. 68% (95%CI: 50–83%) would use it for all their patients and 29% (95%CI: 15–48%) for some of their patients. Only one physician indicated not wanting to use the software at all (3% (95%CI: 0–15%). In free text commentaries physicians appreciated that the software supported their clinical judgement in not always recommending intensive treatment. This appeared to be particular relevant for older patients and those with severe comorbidities, where less intensive treatment may be more appropriate. Overall, physicians felt that patients understood the reasoning behind treatment intensity decisions (median 4, scale 1 to 4). They also believed that patients actively participated in the decision making process median 4, scale 1 to 4).

### Distribution of inputs

**Fig. 2:**
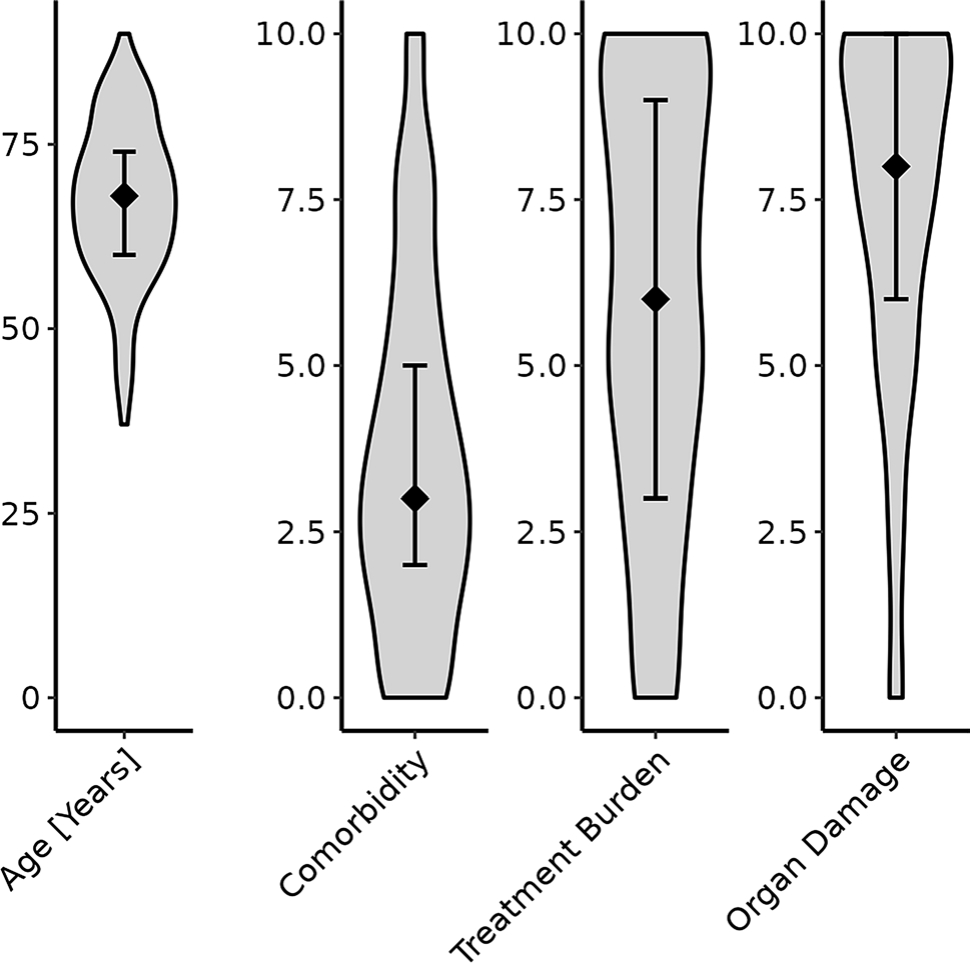
Violin Plots illustrating the distribution of the four patient-specific inputs utilised in “arriba Diabetes”. Inputs include age (median: 68 years [interquartile range: 60–74]), extent of comorbidities (3 [2–5] on a scale 0–10), accepted treatment burden (6 [3–9] on a scale 0–10), and preference to reduce organ damage in the future (8 [6–10] on a scale 0–10)

The inputs age, comorbidities and treatment burden showed a relatively high degree of dispersion. The median comorbidity score was 3 on a 0–10 scale (interquartile range: 2–5) and patients expressed a moderate acceptance of treatment burden (median: 6, interquartile range: 3–9 on a scale 0–10). However, with regard to prevention of long-term organ damage, the median preference of reducing the risk of organ damage in future was 8 on a scale 0–10 (interquartile range: 6–10). Figure 2 illustrates the distribution of the inputs.

### Distribution of outputs

“arriba Diabetes” generated an individualised treatment intensity recommendation divided in three categories. The majority of patients (57%) received a recommendation falling into Category iii (intensive therapy aimed at reducing the risk of future organ complications). Only a small proportion of patients (9%) received a recommendation falling into Category i (prevention of short-term symptoms only).

### Correlation of inputs

**Fig. 3:**
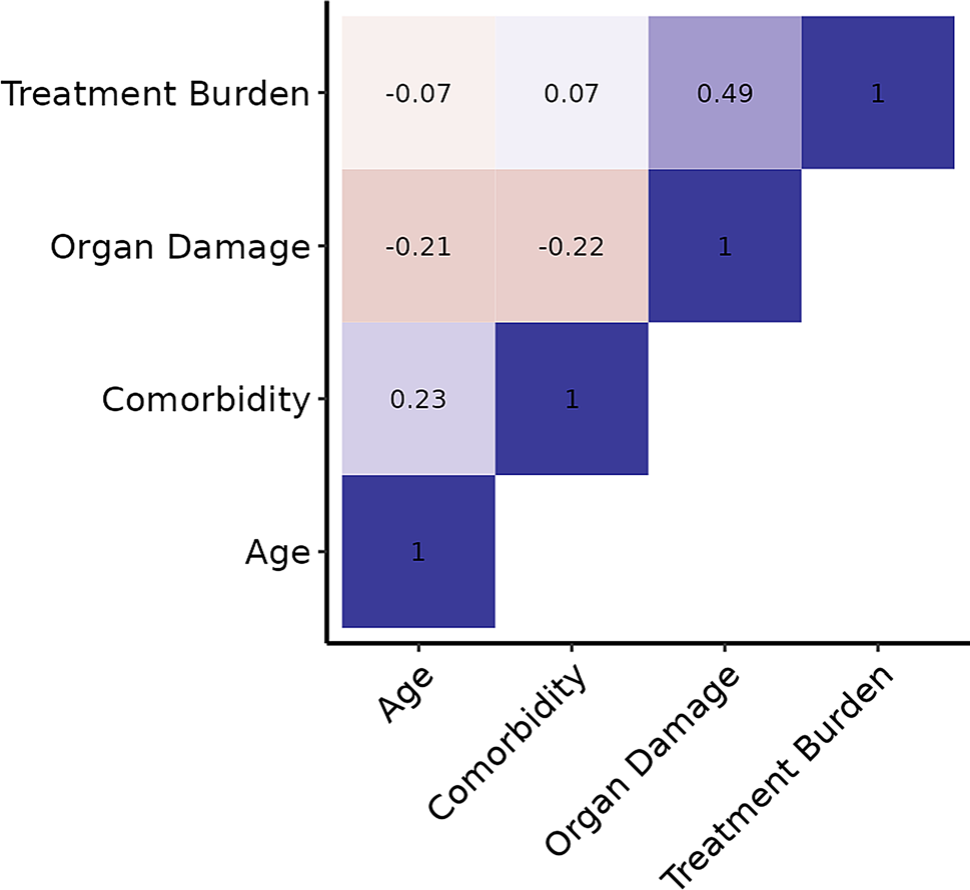
Correlation heat-map showing the correlation of the four inputs. The heat-map visually represents the correlation matrix of the four inputs used in “arriba Diabetes”. Each cell displays the strength and direction of the estimated correlation between two inputs

Next, we investigated correlations among the four inputs. There was a relatively strong positive correlation (Spearman correlation coefficient: +0.49) between the two preference inputs “accepted level of treatment burden” and “preference to reduce future risk of organ damage”. The inputs age and comorbidities showed a moderate positive correlation (Spearman correlation coefficient: +0.23). The input preference to reduce organ damage in future showed a small negative correlation with age and comorbidities (Spearman correlation coefficient: -0.21, respectively − 0.22). Interestingly we did not find a correlation between accepted level of treatment burden and age or comorbidity. Figure 3 displays the correlation heat-map of the four inputs.

## Discussion

Our study revealed a good agreement between the software recommendations for therapy intensity and physicians’ judgements. Study physicians were highly motivated to integrate “arriba Diabetes” in their future care.

### Comparison with existing literature

The utilisation of patient decision aids in diabetes management remains relatively uncommon [6]. Most of existing decision aids in the field focus on specific treatment choices, such as specific antihyperglycaemic drugs or cardiovascular risk calculation in diabetes [7, 8, 22]. Denig et al. developed a decision aid for diabetes patients to prioritize areas of care, such as lipid lowering, blood pressure management, smoking cessation and/or glucose-lowering interventions. However, their highly complex approach built upon quantitative presentations of risk for future morbid events and (absolute) risk reduction, with an emphasis on cardiovascular prevention, presumably not least because of the more reliable evidence base in this area [23]. “arriba Diabetes” is based on a direct elicitation of values, which are fed into a model providing appropriate treatment intensities (HbA1C levels).

The observed negative correlation between the preference for reducing organ damage and both age and comorbidities highlights the importance of incorporating individual patient preference into diabetes care. One potential explanation for this correlation could be that older patients and those with higher comorbidity might prioritise immediate quality of life over long-term preventive measures. Additionally, older patients or those with significant comorbidity may perceive the likelihood of benefiting from long-term risk reduction as limited, given their shorter life expectancy.

A Cochrane review on decision aids for people facing health treatment or screening decisions has shown an increase in the number of patients choosing to initiate new medication for diabetes when these aids are employed [24]. Interestingly, in our study physicians welcomed the use of “arriba Diabetes” to help reducing the intensity of treatment in cases of very old age and frailty. In line with the perspective of physicians in our study, the decision aids summarised in the Cochrane Review also enhance patient participation in the decision-making process [24].

Within health informatics a broad range of knowledge acquisition and computational discovery methods are available. The development of “arriba diabetes” can best be described as “manual” [25]. Interestingly, the literature on this issue assumes a separation of clinical and software engineering expertise, with methods such as interviews, observation or process mining aiming at bridging the gap between the two. “arriba”, however, includes both kinds of expertise within a collaboration lasting for almost 20 years [26].

### Strengths and limitations

An important strength of our study is that we were able to evaluate “arriba Diabetes” in a real life setting, as physicians used the software during their consultation with regular patients, i.e. the setting where it is supposed be used in future.

A strength of the “arriba Diabetes” software lies in its ability to translate patient preferences into actionable treatment goals in terms of HbA1C targets. While HbA1C should not be considered the sole indicator of individualised treatment intensity, its inclusion remains essential as concrete and easily available objective.

It is also important to consider the limitations of the study. Although participating physicians were encouraged to critically evaluate the software recommendation for their patients, there is the possibility that its suggestions exerted some influence on their judgement. In our view, the fact that they expressed their disagreement in a sizable minority of cases suggests that they provided an independent opinion. However, without a control group, it is unclear whether physicians were truly independent in their assessments.

Additionally, it is important to acknowledge the possibility of selection bias among participating physicians. Physicians who engage in research studies without financial incentives may generally be more proactive and enthusiastic towards technologies such as decision support. Furthermore, we only included physicians who were already using the “arriba” software in other circumstances. These physicians might also be more open to a newly developed “arriba” software. This might restrict the generalisability of the findings to the wider primary care population. Furthermore, the potential for selection bias also exists regarding included patients, as the GPs personally approached their patients without specific instructions to include all eligible patients or a randomised sample. This potential selection bias at the level of both physician and patients might lead to an overestimation of the actual agreement rates.

Only physicians were asked to use “arriba Diabetes”. However, we regard it as realistic that other professional groups, such as practice nurses, will apply the software in their diabetes management.

As the study was conducted in an early stage of the software development and evaluation process, we determined the sample size in a pragmatic fashion. The proportion of agreement between software and study physicians was our main outcome. Because the conclusion to be drawn from the study would be identical at the lower (80.4%) and the upper (91.8%) border of the 95% confidence interval, we conclude post-hoc that the study was adequately powered.

Instead of presenting a sophisticated algorithm exploring and weighting each patient’s comorbidities, user physicians are asked to provide a global, intuitive estimation of comorbid load. For this, they were provided detailed instructions including illustrative examples. This approach was well received because as primary care physicians they knew their patients well. This familiarity allowed them to provide quick estimates in a minimum of time.

The specification of the underlying quantitative model could not be derived directly from study evidence. We are not aware of any previous studies quantifying the relevance of preferences and their consequences for management options. Based on health-economic modelling and guideline recommendations, expert judgment is thus crucial to develop a model for treatment intensity recommendation. One consequence, however, is the lack of an objective standard to validate or calibrate the model and its output. While elaborated methodological standards are available for predictive models, this is not the case for models addressing subjective preferences [27]. We therefore chose to validate the decision aid with the expert judgement of primary care clinicians with the individual patient as the unit of observation. This seemed appropriate to us because they combine expertise regarding the natural history and treatment of diabetes with their knowledge of their patients’ histories and characteristics.

The choices we made when specifying our model limit the topics and their weights considered in the consultation. However, users of the software are encouraged to reflect on the appropriateness of the proposed treatment intensity in each case. To facilitate this kind of reflection, the software presents its suggested treatment intensity together with a graphical representation of four input variables. Changes in inputs and their effect on the output thus become immediately visible. Patients and physicians may change input values according to subjectively felt weights. Moreover, they might feel additional considerations to be qualitatively important, such as a patient’s early dementia.

### Implication for research and clinical practice

Our study shows that “arriba Diabetes” software can be integrated in diabetes clinics, providing a practical tool for achieving patient-centred care with individualised treatment targets.

As part of a larger research program, we plan studies to evaluate patients’ voices, including their feedback on being advised with the software and the resulting treatment recommendations. Additionally, we aim to assess the impact on various healthcare processes, such as medication management, the implementation of non-drug measures, and the frequency of referrals to specialists. Furthermore, we want to investigate the feasibility of “arriba Diabetes”, e.g. the duration of consultation and the integration into clinical workflows. Considering the model was developed based on expert opinion, we also plan to further validate the recommendation regarding treatment intensity.

## Conclusions

In summary, “arriba Diabetes” offers a structured approach to defining individual treatment goals and intensity, adressing a long standing challenge in diabetes care. While our findings suggest good alignment with physician judgement, the study does not assess clinical outcomes or broader implementation. Further research is needed to investigate the potential of “arriba Diabetes” to improve care for individuals with T2DM.

## Electronic supplementary material

Below is the link to the electronic supplementary material.

Supplementary Material 1

## Data Availability

The datasets used and analysed during the current study are available from the corresponding author on reasonable request.

## Abbreviations

arriba: Aufgabe gemeinsam definieren (define the task together), Risiko subjektiv (subjective risk), Risiko objektiv (objective risk), Informieren über die Interventionsmöglichkeiten (inform about the intervention options), Bewertung der Möglichkeiten (evaluation oft he options), Absprache über weiteres Vorgehen (agreement on the further plan)
ng: not given
NICE: National Institute for Health and Care Excellence
PC: Primary care
RCT: Randomised controlled trials
SDM: Shared decision making
