# Supplementary material for: Assessing physicians’ agreement and the completeness of the decision aid ‘arriba Diabetes’: a cross-sectional study
Source: BMC Prim Care. 2025 Jul 8;26:218. doi: 10.1186/s12875-025-02887-x (PMC12235830; doi:10.1186/s12875-025-02887-x)
Supplement: Supplementary file 1 — Supplementary Material 1 [file 12875_2025_2887_MOESM1_ESM.docx]

# Supplements

**Supplement 1:**

**Overview of the „arriba Diabetes” software**

Originally the software is German. For publication we have translated the software into English.

| 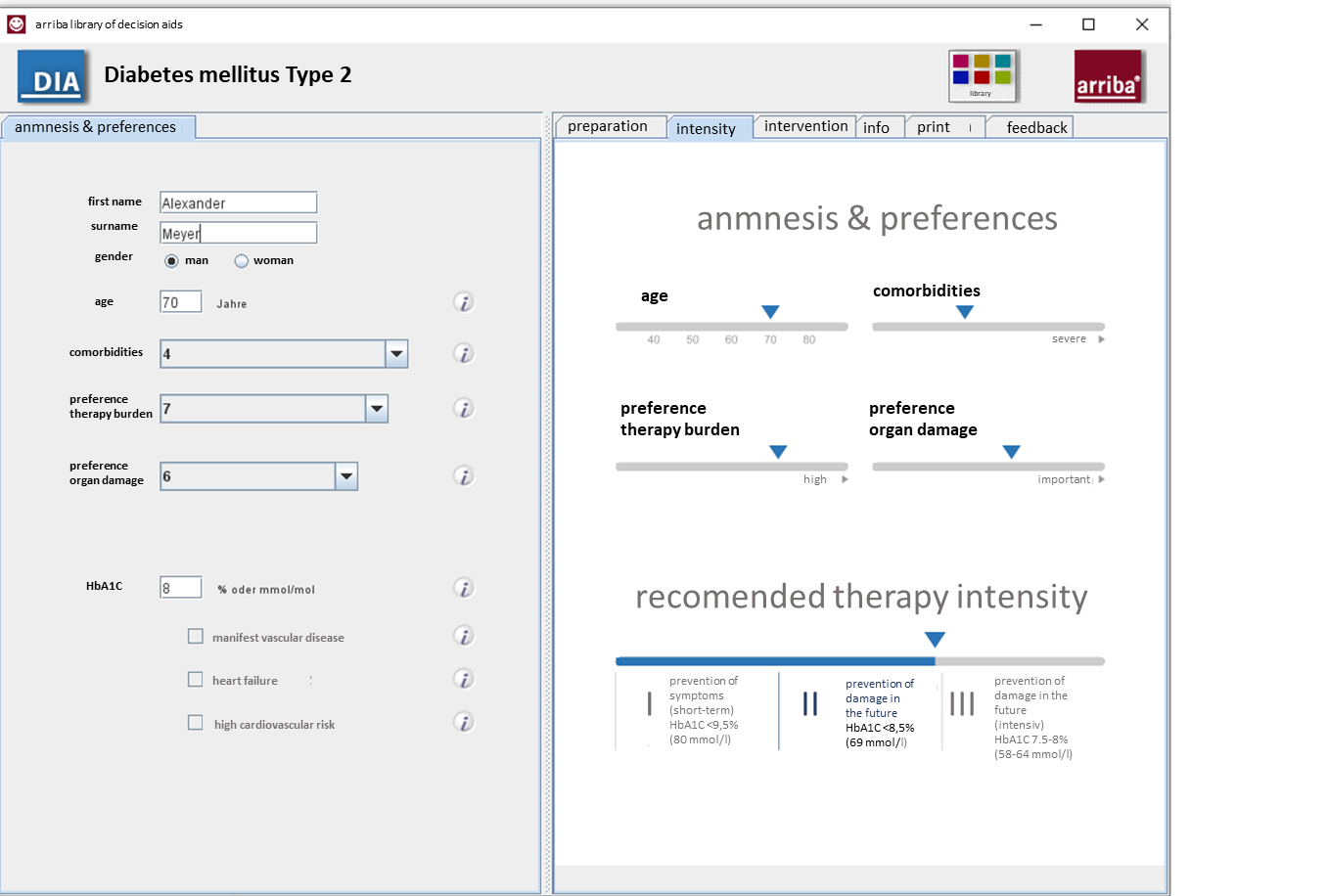 |
| --- |

**Supplement 2**

**Case report form**

| 1 | Patient demographic and medical data:  Gender:   - male - female - diverse   How long is the diabetes diagnosis known?   - < 5 years - 5-10 years - > 10 years   Currently prescribed diabetes medication (multiple entries possible):   - Biguanides, (e.g. metformin) - Sulphonylureas, (e.g. glibenclamide, glimepiride, gliclacid) - Gliptins, (e.g. e.g. sitagliptin, saxagliptin, vildagliptin) - GLP-1 analogues (e.g. exenatide, liraglutide, albiglutide, dulaglutide) - Gliflozines as SGLT2 inhibitors (e.g. dapagliflozin, empagliflozin, canagliflozin) - Insulin - Other: _________________________________________ |
| --- | --- |
| 2 | **Do you think the recommended therapy intensity is appropriate for your patient?**   - yes - no   If no:  Please mark the therapy intensity you feel is appropriate on the bar:  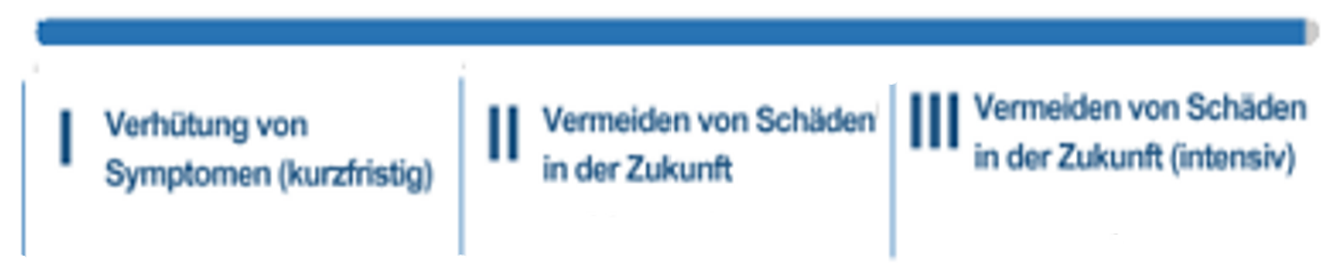  = I Prevention of symptoms II Avoiding complications in III Avoiding complications in  (short-term) the future the future (intensive) |
| 3 | **Do you consider the therapy suggestions (‘interventions’) to be appropriate?**   - yes - no   If no: Please indicate alternative (preferable) suggestions:  ______________________________________________________ |

| 4 | **Do you have the impression that your patient has understood the decision about the intensity of treatment?**  no yes   \| **1** \| **-** \| **2** \| **-** \| **3** \| **-** \| **4** \| \| --- \| --- \| --- \| --- \| --- \| --- \| --- \|   **Do you have the impression that your patient was able to contribute to the decision on treatment intensity?**  no yes   \| **1** \| **-** \| **2** \| **-** \| **3** \| **-** \| **4** \| \| --- \| --- \| --- \| --- \| --- \| --- \| --- \| |
| --- | --- | --- | --- | --- | --- | --- | --- | --- | --- | --- | --- | --- | --- | --- | --- |
| 5 | **Will you use the arriba module when it is available?**   - yes - no   If no: What are your concerns?    ______________________________________________________ |
| 6 | **Any other comments?**  **For example on the layout, i-buttons and info texts, or on the brochure?**  ­­­­­­­­­ ______________________________________________________ |
